# Using GIS Remote Sensing Image Data for Wetland Monitoring and Environmental Simulation

**DOI:** 10.1155/2022/7886358

**Published:** 2022-08-29

**Authors:** Qian Cheng, C. N. Dang

**Affiliations:** ^1^College of Water Conservancy, Shenyang Agricultural University, Shenyang, Liaoning 110866, China; ^2^Ho Chi Minh City University of Transport, Ho Chi Minh City, Vietnam

## Abstract

Through a comprehensive theoretical basis and actual test analysis of the application system design and functional efficiency of the cloud platform, this paper puts forward an artificial intelligence environmental data monitoring and wetland environmental simulation method based on GIS remote sensing images. First, the basic storage and computing functions have been enhanced at the physical layer. Second, the middleware layer is more flexible in the use of management methods and strategies. There are many strategies and methods that can be used in combination. Finally, based on this, the application system design framework is more convenient and faster so that you can focus on business logic, and the strategic advantages of certain functions are very obvious. The method of object-oriented classification and visual interpretation using UAV image data and satellite remote sensing images from the typical recovery area and treatment area of wetland from 2016 to 2020 is given in detail together to extract wetland information and use GIS software for dynamic calculation. Using the wetland transmission matrix method, the distribution map of the characteristic types of the survey areas in the four periods and the conversion status of the characteristic types at each stage were obtained, and the effect of wetland treatment was quantitatively studied.

## 1. Introduction

Due to the recent social development, rapid economic development, and increase in pollutant emissions, the environment of the estuary wetland has gradually become bad. Therefore, in order to simulate the changes in the ecological environment of the wetland in the estuary area, a simulation model of the wetland environment was established [1]. In order to facilitate the dynamic simulation of the model and the various changes in the development plan, predict the future development direction of the wetland ecological environment, and reveal the status and influencing factors of the wetland ecological environment in various future development scenarios [2]. In-depth understanding of the relationship between the environment and social and economic development and then to solve the ecological and environmental problems of the wetland, but also to have a certain fundamental basis for the sustainable development of the site [3]. This paper first gives a series of introductions to the research significance of the thesis and the status quo at home and abroad [4]. Then, based on the content of the survey, the overall system structure of the organization and the content to be researched are roughly designed and organized, and improve the theoretical concepts and content related to GIS remote sensing images, and make the theoretical basis of GI remote sensing image ecological environment data monitoring more stable [5]. According to this model, four ecological environment change plans are designed, each of which uses the constructed model for simulation and prediction and combines artificial intelligence technology to provide a better plan for future development [6]. The simulation results of the simulation model show that the key factors that will have a great impact on the ecological environment of the wetland are the area of the investigated wetland, the water quality of the wetland, and the amount of water required for the ecological environment [7]. If a series of developments continue in accordance with the current environmental model, wetlands will show a downward trend in the future. The health and sustainable development of the wetland ecosystem can only be maintained through a series of changes in policy measures, such as water saving and emission reduction [8]. Through the dynamic simulation of the estuary wetland ecological environment, it can reflect the natural and social indicators of the estuary wetland ecological environment and also clarify the feedback relationship between the screening indicators, and a complete indicator system has been installed [9]. Finally, according to the biological seasonal research of the main plants of reeds and wetlands, as well as through outdoor observation, visual interpretation of drone image data in various seasons and spectral information of satellite images, and so on, the classification accuracy verification is carried out [10]. The final result proves that autumn is the best season for reed wetlands to resume monitoring [11].

## 2. Related Work

The literature introduces the scientific and reasonable classification of wetlands. The complexity and uncertainty of the phenomenon of “different spectrums with the same quality” and “different spectrums with the same spectrum” in the spectral information of wetland vegetation, as well as the inherent complexity and unknown characteristics of the studied wetlands, form the basis for the extraction of remote sensing image information [12]. The original wetland monitoring classification technology was combined with other data sources for comprehensive analysis, mainly based on artificial visual interpretation of images [13]. Chinese scholars Wen Ming and Wang Minhua proposed a scheme called image landscape ecology based on the current specific situation and extracted five landscape ecology models based on the visual interpretation of Dongting Lake wetland plants [14]. Since visual interpreting is a highly flexible and highly dependent interpreting method, interpreters have high requirements for long-term and arduous tasks. This is only suitable for extracting smaller spatial scale information [15]. The literature introduces a remote sensing technology for drones, which is mainly based on drones to build this aerial platform and then uses remote sensing sensors or camera images for preprocessing when acquiring ground-related object information; a technology for stitching images according to specific requirements is introduced [16]. The key advantages of the system are that the development cost required to study the system is relatively low, the cycle required for the system is relatively short, and the system has high repeatability, small size, simple operation, and high safety. Most importantly, we can retrieve high-resolution remote sensing image data suitable for local research projects in our area. The literature introduces the remote sensing monitoring database of the reed wetland in the estuary. The database consists of remote sensing data and measurement data [17]. Remote sensing data includes aerial photography data from drones and satellite images of Gaofen-1 and Ziyuan-3. The measurement data includes field survey data classification result verification point data. The literature introduces the preprocessing analysis of satellite imagery [18]. Based on the results of on-site verification and visual interpretation of UAV image data, we use object-oriented classification and a series of trials and errors to determine the scale with the best segmentation effect for various image objects [19]. This is to achieve the purpose of evaluating classification rules according to levels, as well as the accuracy of classification execution results, and extracting wetland information. The literature introduces system dynamics using cybernetic technology, which is not only widely used in social and economic systems but also widely used in the field of environmental research at home and abroad [20]. It provides certain scientific and theoretical decision-making basis for the planning schemes and implementation actions of a series of related projects such as urban land and environmental planning, urban and river basin water resources, and environmental capacity. The literature introduces the reading and preprocessing of remote sensing image data. With the emergence of various types of remote sensing image data, the system can meet the needs of new data support and convert the new data into a unified format that can be recognized by the platform. The system should also have the function of preprocessing remote sensing data (atmospheric correction, in situ correction, cloud detection, etc., within the jurisdiction). Preprocessing is the first step in applying remote sensing data; otherwise, the extracted subject information will be meaningless.

## 3. GIS Remote Sensing Image Environmental Data Monitoring and Environmental Simulation Technology

### 3.1. GIS Remote Sensing Image

#### 3.1.1. Data Preprocessing

**Figure 1:**
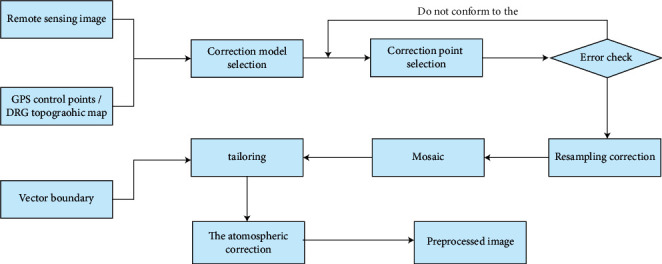
Flowchart of remote sensing image preprocessing.

The preprocessing of remote sensing images is divided into a multistep process. The process includes image embedding, merging, cropping, cloud removal, atmospheric correction, and geometric correction. The geometric correction also includes geometric precision correction, accurate image matching, correctness, and radio correction. The remote sensing image is a medium-resolution multispectral image, and its preprocessing process is shown in Figure 1.

Radiation inspection is the process of converting the DN value recorded by the sensor into an absolute radiance value. Generally, the radiation correction formula is

(1) $$\begin{array}{c} \text{Radiance}=\text{Gain}\times \text{DN}+\text{offset.} \end{array}$$

The China Resources Satellite Center network provides correction factor correction formulas for satellite data of other countries/regions. Among them, there are some differences between the HJ-1 A/BCCD radiation calibration formula and the GF-1/WFV formula. The specific formula is

(2) $$\begin{array}{c} \text{Radiance}=\frac{\text{DN}}{\text{Gain}+\text{offset }}\text{.} \end{array}$$

#### 3.1.2. Data Sheet Design

Through the analysis of the main technologies of remote sensing data integration technology, the content of metadata and the actual needs of the system are summarized, and a database table containing image tables, metadata tables, and theme-specific product tables is designed. Some examples of database tables are given in Table 1.

The satellite information is shown in Table 2.

The sensor information is shown in Table 3.

The remote sensing clever image is shown in Table 4.

The metadata information is shown in Table 5.

#### 3.1.3. Information Extraction

Some researchers established an inversion algorithm based on the sky blue data actually measured in the Yellow Sea and the East China Sea, using the sky blue component of China's first ocean color satellite (HY-1A) CCD imager. Others went to the Yellow Bay area and used H-star CCD data to assess water quality, and the results were consistent with the actual situation. Therefore, the reversal algorithm of this module can use the existing correlation results, as shown in the following:

(3) $$\begin{array}{c} Chla=-0.1961x^{3}+2.6833x^{2}-11.903x+17.953,\\ x=\left(\frac{R_{rs}\left(461\right)}{R_{rs}\left(566\right)}\right)*{\left(\frac{R_{rs}\left(652\right)}{R_{rs}\left(566\right)}\right)}^{-0.62}. \end{array}$$

When the suspended sediment concentration SSC> 5 mgL, it is a medium-high turbid water body:

(4) $$\begin{array}{c} Chla=474.81x^{3}-461.41x^{2}+44.008x+37.95,\\ x=\left(\frac{R_{rs}\left(461\right)}{R_{rs}\left(566\right)}\right)*{\left(\frac{R_{rs}\left(652\right)}{R_{rs}\left(566\right)}\right)}^{0.3}. \end{array}$$

Total suspended particulate matter is an important parameter for monitoring the environmental quality of coastal waters in China. Affected by the source of the land, suspended solids often carry some pollutants and cause water pollution. In order to reverse the concentration of suspended solids near the coast, this paper adopts the Hangzhou Bay suspended solids model. The formula is

(5) $$\begin{array}{c} \text{TSM}=10^{1.0758+1.1230*\text{Ratio}},\\ \text{Ratio}=R_{rs}\frac{\left(745nm\right)}{R_{rs}\left(490nm\right)}. \end{array}$$

For the vegetation of winter wheat, the correlation between the biomass and the vegetation index created by Xu and others has been recognized. They found that it has a greater correlation with the NDVI index. The calculation formula is as follows:

(6) $$\begin{array}{c} BI=116.7{\text{e}}^{1.638^{*}\text{NDVI}}. \end{array}$$

For the biomass of rice, the calculation formula is as follows. This formula was proposed by Hu et al.

(7) $$\begin{array}{c} B2=87.0518+106.5892\text{NDVI.} \end{array}$$

The calculation formula for biomass energy evaluation is as follows:

(8) $$\begin{array}{c} E=B\times R\times \lambda \times H. \end{array}$$

According to the evaluation results of straw biomass energy, subtract the conversion factor used as propagation material (i.e., the factor that can be actually used), and the crop straw biomass energy can be developed and utilized.

(9) $$\begin{array}{c} Q=E\times \varphi . \end{array}$$

User accuracy (UA) refers to the ratio of a specific type (the *i*-th row of the confusion matrix) to the total number of pixels correctly classified for that type. This means that the result of this classification can represent the true proportion of types. The formula is

(10) $$\begin{array}{c} {\text{p}}_{ij}=\frac{x_{ii}}{x_{+j}}. \end{array}$$

Producer accuracy/graph accuracy (PA) is the percentage of a sample (column *j* of the confusion matrix) of the actual measurement type of a certain type of reference classification data. The formula of producer accuracy/graph accuracy (PA) is

(11) $$\begin{array}{c} {\text{p}}_{ji}=\frac{x_{ii}}{x_{i+}}. \end{array}$$

The total accuracy (OA) is the ratio of the number of correct classifications to the sum of the total reference data (only diagonal pixels) during the sampling process, reflecting the accuracy of the total classification results. The total accuracy (OA) is described as follows:

(12) $$\begin{array}{c} p_{n}=\frac{\sum_{i=1}^{n}x_{ii}}{N}. \end{array}$$

The Kappa coefficient most accurately reflects the accuracy of the overall classification. This is based on each data type in the confusion matrix as follows:

(13) $$\begin{array}{c} K=\frac{N\sum_{i=1}^{n}x_{ii}-\sum_{i=1}^{n}\left(x_{i+}\times x_{+j}\right)}{N^{2}-\sum_{i=1}^{n}\left(x_{i+}\times x_{+j}\right)}. \end{array}$$

### 3.2. Environmental Simulation Technology

#### 3.2.1. Mathematical Principles

System dynamics is an academic research based on cybernetics, in which the system is treated as a description of a differential equation. Therefore, a set of differential equations can also be used to express this system. Among them, nQ represents some quantitative attributes of some elements of the system.

(14) $$\begin{array}{c} \frac{\text{d}Q_{1}}{\text{d}t}=f_{1}\left(Q_{1},Q_{2},\ldots ,Q_{n}\right),\\ \frac{\text{d}Q_{2}}{\text{d}t}=f_{2}\left(Q_{1},Q_{2},\ldots ,Q_{n}\right),\\ \frac{\text{d}Q_{n}}{\text{d}t}=f_{n}\left(Q_{1},Q_{2},\ldots ,Q_{n}\right). \end{array}$$

If the stock represents a certain variable Y, its flow represents the difference of *Y* over time (*T*). This is the amount of change in Y over time. If the differential equation is known,

(15) $$\begin{array}{c} \frac{\text{d}Y}{\text{d}T}=Y+X. \end{array}$$

The mathematical expression is

(16) $$\begin{array}{c} \frac{\text{d}Y}{\text{d}t}=\frac{Y\left(t\right)-Y\left(t-\text{d}t\right)}{\text{d}t}\Rightarrow Y\left(t\right)=Y\left(t-\text{d}t\right)+\frac{\text{d}Y}{\text{d}t}\text{d}t. \end{array}$$

#### 3.2.2. Quantification Method of Wetland Environmental Indicators

The water environment quality, wetland area, and the satisfaction of ecological water demand belong to different dimensions, which are three indicators of the ecological environment quality of estuary wetland. Therefore, taking the ecological system medicine method as a reference, the environmental quality evaluation index system of the estuary wetland ecosystem is summarized into a three-element structural system, and the coefficient of variation is used to determine the respective weighting methods. We use national indicators and scope laws for standardization. Through the establishment of a comprehensive evaluation model of the estuary wetland ecological environment quality index, the environmental quality of the estuary wetland ecosystem is characterized.

For the bigger the healthier index:

(17) $$\begin{array}{c} \bar{X_{ij}}=\frac{X_{ij}-X_{j\min}}{X_{j\max}-X_{j\min}}X_{ij}\in \left[0,1\right]. \end{array}$$

For the smaller the healthier index:

(18) $$\begin{array}{c} \bar{X_{ij}}=\frac{X_{j\max}-X_{ij}}{X_{j\max}-X_{j\min}}X_{ij}\in \left[0,1\right]. \end{array}$$

Among these three indicators, it cannot be used for direct analysis because the percentage of contribution to the ecological environment indicators of the Liaohe Estuary wetland is different, so the weight of each indicator must first be determined.

(19) $$\begin{array}{c} \delta_{j}=\frac{D_{j}}{\bar{X_{j}}}. \end{array}$$

Index weight calculation is as follows:

(20) $$\begin{array}{c} A_{j}=\frac{\delta_{j}}{\sum_{j=1}^{n}\delta_{j}}. \end{array}$$

## 4. Wetland Environmental Data Monitoring and Wetland Environmental Simulation Research

### 4.1. Overview of the Study Area

**Figure 2:**
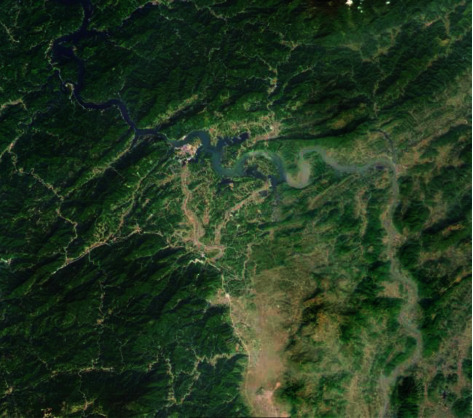
Geographical location map of the study area.

The estuary reed wetland is derived from 21 estuary water systems (including Taitung River, Liaohe River, and Daling River) after a long period of evolution, and the sediments in them gradually developed naturally. The research field of this paper is located in S River Nature Reserve. This is a representative reed wetland restoration area with an area of about 4000 km^2^. Refer to the geographic location map for details, and the study area is shown in Figure 2.

#### 4.1.1. Topography

The surveyed area is an alluvial floodplain of the Lower Mainland, located in the southwest of the L Delta, with a single topography. The terrain in the north is higher, and the proportion in the south is reduced to about 1/10,000. The height of the swamp is 1.7–3 m.

#### 4.1.2. Climate

The estuary wetland area is geographically located in an area biased to midlatitudes, and most of the area in that range is a temperate continental monsoon climate with a warm temperature nature. The four seasons are sunny, and the temperature is moderate, which can meet the growing demand of major crops such as reeds. In spring, it warms rapidly due to strong winds, little rain, and a lot of evaporation. Winter is cold and dry, with almost no precipitation. The coldest month is January, and the actual temperature may reach −29.3 °C. The catastrophic weather in the survey area included heavy rain, strong wind, and frost.

#### 4.1.3. Hydrology

In the estuary wetland, the surface water system is developed. The mountain river and L river are mainly used for drainage. Before the wetland restoration and treatment project, the irrigation of the reed field mainly depends on the D river and the mountain river. The mountain river is affected by the tide, and the river water itself has high mineral content. There are two flood seasons in spring and summer in the Panjin area. The spring flood season is short, water flows and fresh seawater are introduced from the reed field irrigation reservoir, and the summer flood period is long, which is prone to floods and heavy rainfall. When a disaster occurs, heavy rain will cause reeds to grow, and when the salinity of the irrigation water is low, many weeds, such as cat tails and water onions, will appear in the reed wetlands.

#### 4.1.4. Soil

The main soil types of estuary wetland are paddy soil, saline soil, pasture soil wetland, and oil sand soil. Among them, paddy soil forms the largest area in the long-term hydroponic maturation of human activities and is the most widely distributed area, accounting for 40% of the total soil area. Saline soil is due to wetlands covered by water for many years. Due to the slow decomposition of nutrients, the soil is poorly ventilated and has high salinity. Wetland soil is permeable soil, distributed in lowlands by rivers, banded reed swamps, or depressions on plains, accounting for 8.5% of the total area. Sandy soil is partly distributed in the western and northeastern regions. The main soil types of reed wetland studied in this paper are pasture soil and wetland soil.

#### 4.1.5. Natural Resources

The L estuary reed wetland has a huge area, and therefore, it is the largest estuary reed wetland in Asia. In the estuary wetland, reed is a relatively important community species. The coverage rate of the entire community exceeds 90%, and only some companion species, such as cattail grass and calcarina, are distributed in the lower layer. Due to its unique natural wetland landscape and abundant animal resources, the estuary wetland is a habitat and habitat for migratory birds. According to long-term observations, there are major national protected animals, such as cranes, swans, and other rare species. The tidal flat area on the south coast of the estuary wetland is 39,200 hectares, and the reserves of water resources such as fish, shrimps, and crabs are about 40,000 to 50,000 tons, accounting for 70% of the total reserves of L Dongwan.

Reed is an important building species in the estuary wetland. Estuary reed marshes are mainly distributed in the D River port in the north of the Dal estuary. There are mainly six reeds: A reed field, B reed field, C reed field, D reed field, E reed field tributary, and F farmland. Most of the reed groups are distributed in freshwater wetlands with accumulated water for many years or a certain seasonal accumulation of water. The community is divided into swamp reed and pasture reed communities. Forage reeds are saline-alkaline soil, and the total coverage of reeds in wetlands exceeds 90%, which is absolutely dominant. According to the military maps and remote sensing image analysis and investigation of the L estuary area from the late Ming to the early Qing, the formation of the coastal reed wetland at the L estuary was in the Qing Dynasty, and the history of its formation and evolution is only over 100 years. Many years ago, the period from 1909 to 1945 was a period of rapid formation of coastal reed wetlands. The naturally silted reed wetlands along the L River Delta extend to the sea. As a highly fire-resistant plant, *Suaeda pterocarpus* has become a pioneer species that forms a large number of communities in the intertidal zone, and it also has a desalination effect. Tidal changes deprive the soil of salt and inhibit the return of salt. It provides salt to the soil and therefore makes the soil deteriorate, suitable for the growth of reed plants. In 1995, in the past 20 years, the extent of the wetland completely formed by the coastal reed marshes at the mouth of the L estuary has hardly changed. Recently, the reed wetland at the mouth of the L River has been facing the threat of degradation. In the 30 years from 1984 to 2013, the proportion of natural wetland area in the L estuary dropped sharply, and the value was once reduced from 121690.18 hm^2^ in the previous survey to 97344.66 hm^2^ in the previous survey. At the same time, freshwater limits the changes in salinity. Therefore, freshwater resources are scarce, which is the main factor limiting the production of reeds. In recent years, the climate in the delta has been warm and dry, and freshwater resources have been declining. This can be demonstrated by uneven precipitation and a significant reduction in natural runoff. At the same time, the number of artificially fertilized rice fields has increased, and the lack of water in reed fields has increased. In order to prevent the infiltration and irrigation of tidal water, tidal gates have been built in various algae passages in reed fields. Due to lack of water, the area of reeds can hardly reach 80,000 km^2^. If irrigation is insufficient, reeds will become scarce, and their height will decrease. The degradation of reed wetland has seriously affected the economy of Panjin area and, more importantly, affected the ecological and environmental functions of the reed wetland in the Liaohe River.

At the same time, the development and construction of the L River Oilfield crossed the oilfield roads, the water collection and transportation pipelines extended in all directions, and the oil production facilities were full, causing great damage to the irrigation and drainage system in the area. Large patches of pampas grass grew in the reed field, indicating the degradation of the reed marsh. The survival and growth degree and quantity of animals and plants in reed wetlands are gradually decreasing, which is caused by the destruction of natural ecological environment such as water bodies, topography, and soil in reed wetlands.

### 4.2. Data Source

A total of 12 remote sensing images were collected from the L River Estuary Reed Wetland Research Area. It includes the key research data of the resource-3 satellite remote sensing image in 2018 and the Gaofen-1 satellite remote sensing image in 2017, 2019, and 2020 (see Table 6). During this period, there were 6 field visits to survey data in the survey area. Among them, the remote sensing images of Gaofen-1 and Ziyuan-3 were purchased from a remote sensing image company. The drone image was taken from outdoor aerial photography by Li Jiacun and Deng Lei from the Resource and Tourism College of a certain university.

### 4.3. Monitoring and Analysis of Wetland Environmental Data

The main difficulty in extracting plants with high spatial resolution lies in “the same material with a different spectrum and the same spectrum as the foreign matter.” Wetland plants have different seasonal spectral reflectance characteristics of the same plants; the cattail grass grows near water bodies and has a long growth cycle, reaching its peak only in September. For the summer when plants are more active, the range will be expanded, and the spectral information of various green plants is very similar. The former uses the special “trilateral” parameters of plants to classify plants with similar spectral reflectance, but because this paper only uses 4 image data bands, especially without red edge bands, the vegetation type is relatively accurate only from the image. The classification is not rigorous.

**Figure 3:**
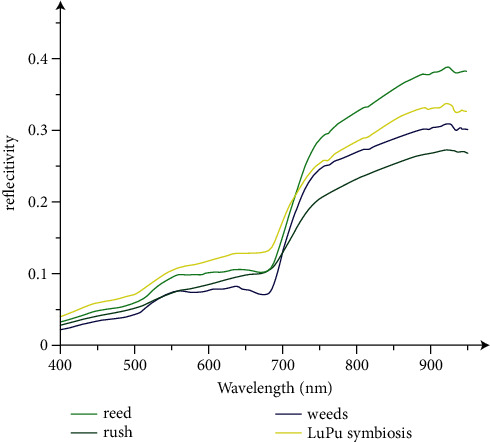
Spectral curve of main vegetation in Liaohe Estuary in autumn.

Autumn is mainly the color change period of reeds and rushes and the season when the spectrum changes significantly (Figure 3). As photosynthesis decreases, the chlorophyll content decreases, and the color of the image gradually changes to yellow and brown. In autumn, the reed is in full bloom, and its reflectivity in a certain band becomes lower than before, but its overall brightness value is indeed higher than before. In autumn, the leaves gradually change color from areas farther from the roots to the roots. Even if the growth period is long, the color of the top of the plant begins to change, and the color of the top becomes light brown, covering the green part of the bottom, and the overall hue becomes darker than that of the reed plant. At the same time, both plants are in the fall of autumn, and the density of the plants is decreasing, which makes them easy to distinguish.

**Figure 4:**
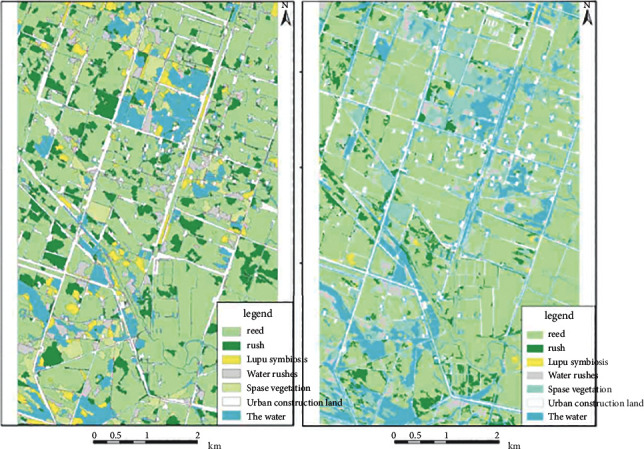
Comparison of seasonal classification results of satellite images.

The results of image analysis and classification in August 2019 (Figure 4) show that cattails have begun to merge and grow after classification, and it is obvious that the areas of cattails that have been removed in the past few years also show that they are overgrown with cattails or Lupu symbiosis areas. Particularly in the top center of the image, cattails generally grow near water bodies, but the analysis in August showed that cattails that were more mature than the previous year did not appear in the waterways and appeared in the reeds that did not follow the wetland growth method. However, the interpretation results in November 2017 have obviously existed since last year. The area of marsh plant reeds has gradually decreased, and the pampas grasses have changed from poor growth to withered. According to this, reeds will grow after 1-2 years, and the growth of *Phragmites australis* accords with the law of wetland restoration in two years.

### 4.4. Monitoring and Analysis of Wetland Types

**Figure 5:**
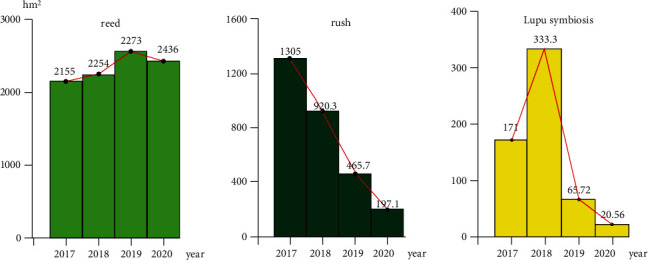
The change trend of typical features in the study area.

As a general experience of estuary wetlands, the overall spatial distribution of reeds has always been a relatively wide area for this area. During the four years of development, the total area of reeds has increased by approximately 281.01 km^2^ compared with the previous survey (Figure 5(a)); after a series of treatments, the distribution range of rushes in recent years There is an obvious downward trend. The overall distribution area has decreased by 1108.01 km^2^ compared with the previous one (Figure 5(b)). There is a certain conversion ratio between the reed and the reed. For the distribution area of Lupu symbiosis, the distribution area first increases. The downward trend (see Figure 5(c)). This indicates that the vitality of the reed is weakened after treatment. After the death of a large piece of pampas grass, new vegetation has not yet grown. In 2017, the pampas grass types that died began to appear in certain areas; water enters the reed fields to treat wetlands. Due to the large amount of water, the overall area covered by water has also increased. Its area roughly increased by 388.31 km^2^.

2019 is the background data studied in this paper, which represents the spatial distribution of wetlands before processing and restoration. The ratio of wetland land features is reeds > shrubs > urban construction land > ocean > symbiosis of urgently sparse plants > sparse plants. The classification results show that platinum stone accounts for 31.65% of the survey area. Although it is less than that of reeds, platinum stone grows in a large area, and patches are found in the reed wetlands. The roots of platinum are mainly grown in water. Below 30 cm, it is more suitable for growing in water than reeds, and grasses are mainly distributed near water bodies. 2017 is the initial stage of the seawater fountain and irrigation project, and the well platform road treatment project and irrigation drainage system have been completed. It can be seen from the classification results that the characteristic weight of wetland is reed > grass > symbiosis of reed and grass > urban construction land > ocean > sparse vegetation. Cattails have been demolished on a large scale, but the area of reeds has not changed much. The reason is that although the cattails have not been completely removed, the leaves are yellow because they coexist and deteriorate. This part of the area accounts for 8.09% of the total; on the other hand, weed control mainly uses the high salt content in seawater, and cattail is not salt-tolerant but withered. Although it has a certain degree of salt tolerance, the rapid increase in salt concentration in the reed field will undoubtedly affect the growth of the reed and the increase in the area of the reed. The characteristic weight of wetland in 2018 is reed > peak > ocean > urban construction site > dead > peak symbiosis > sparse plant. Although this is the most obvious period of change after the restoration of the wetland in 2019, the cattails are still widely distributed and dispersed. Continuous seawater irrigation can prevent these sturdy residual cattails from recovering and growing. In 2019, when the foundation of the treatment project is completed, the effect of wetland restoration becomes more obvious. The ratio of wetland types is reed > water > urban construction site > sparse vegetation > dead cattail > cattail > Lupu growing together. Field investigations showed that there were almost no spots on the kiln. The distribution of kilns only accounted for 5.06% of the survey area, a decrease of nearly 85% compared with 2017. At the same time, it is obvious that, in 2016, the range of reeds decreased slightly, and insufficient growth of wild reeds was observed. This is because seawater irrigation makes the salt concentration in the reed field too high. The weather conditions in Panjin have been harsh in the past year. The growth situation is deteriorating, but field investigations show that the proportion of reeds is increasing, and the yield per unit area is increasing. It can be clearly seen from the image classification results that the area of water bodies has increased compared with the previous year. Due to the large water bodies, some areas have sparse vegetation, and this category is distinguished by images classified as tribal plants, which accounted for the total amount of 7.12% in 2019.

### 4.5. Wetland Environment Simulation Results

**Figure 6:**
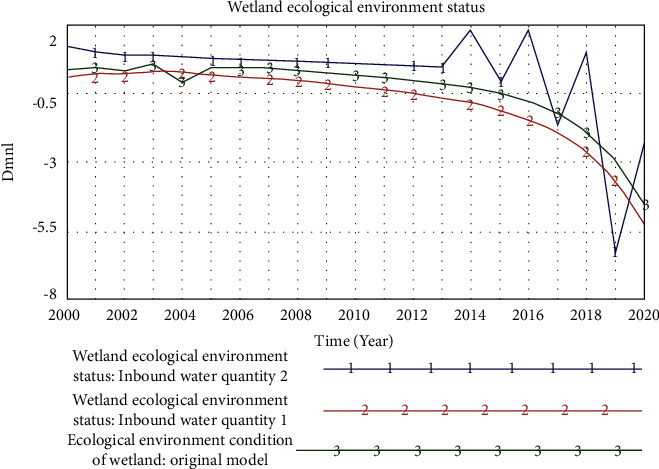
Model sensitivity analysis.

The sensitivity to model value changes should be low. Since there are many parameters suitable for the ecological environment of the estuary wetland, we choose representative parameters. Here, we make assumptions for 2020 and then use this assumption to test the model (see Figure 6). Among the important parameters of the system, the inbound flow of the Liaohe River in Panjin City was selected. This parameter has a great impact on the ecological environment of the wetland. In this figure, inflow 1 means that the inflow in 2020 is expected to be half of 2012, and inflow 2 means that the inflow in 2020 will be twice that in 2012.

**Figure 7:**
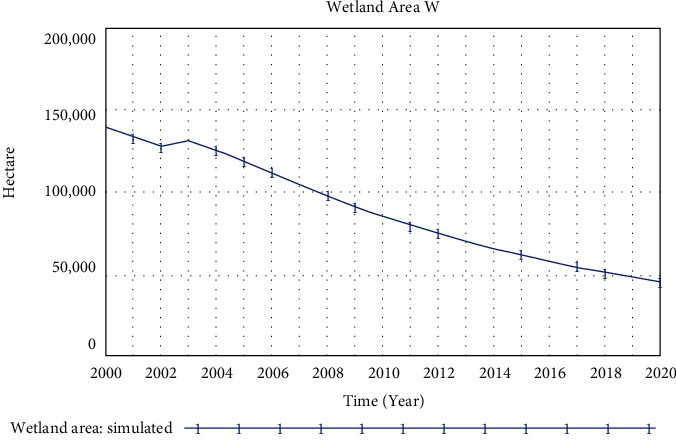
Simulation results of wetland area indicators in the estuary area.

A simulation of the current development status shows that the wetland area has been reduced from 128,000 hectares in 2000 to 56,992 hectares in 2015 and 42,126 hectares in 2020, which is less than half of that in 2000 (see Figure 7).

## 5. Conclusion

Due to the complexity of the current ecological environment problems, the existing 3S technology is difficult to meet the requirements of real-time monitoring and evaluation of the ecological environment. GIS remote sensing images have a new method to solve this problem. In this regard, this paper focuses on the theoretical content of GIS remote sensing images in the ecological environment data monitoring and evaluation, mainly to organize and improve the content of GIS remote sensing in the field of ecological environment, the relevant framework of its technology and the industry, the development direction of Hadoop, the created system applications, and the corresponding patterns of the applications; through the research and application of the Hadoop open-source architecture and its key technologies, the basic environment of GIS remote sensing images is constructed on the basis of the Hadoop architecture. Model technology is used to effectively store and manage large amounts of remote sensing data. The Hadoop framework is used to store and manage a large amount of spatial data. Based on GIS remote sensing images, an ecological environment data monitoring and evaluation application system is constructed. Design artificial intelligence based on the business logic of ecological environment data monitoring and evaluation. The functional modules of the application system analyze the parallelism of each function and combine strategies to complete the application system development; the ecological environment monitoring and evaluation application system realizes the reliability and execution efficiency of each function, analyzes the effective storage of massive spatial data and management technology, and compares it with conventional independent commercial GIS remote sensing images. Finally, a simulation model of the estuary wetland environment simulation was established.

## Figures and Tables

**Table 1:** List of database tables.

Table name	Description
TB_SATELL1TE	Satellite-type information
TB_SENSOR	Sensor-type information
TB_BAS_Tiff	Remote sensing image data table
TB_BAS_MEIADATA	Satellite data metadata information
TB_REMOTEPRODUCTTYPE	Remote sensing product classification information
TB_PRODUCT	Special product information sheet used to record the products that the platform has produced and stored in the warehouse
TB LOG	Operation log

**Table 2:** Satellite information table.

Table name	Type of data	Annotation	Constraint
SatellitelD	INTEGER	Satellite-type automatic numbering	Primary key
SatelliteName	VARCHAR(20)	Satellite name	
Introduction	Text	Satellite introduction	

**Table 3:** Sensor information table.

Table name	Type of data	Annotation	Constraint
SensorlD	INTEGER	Sensor-type automatic numbering	Primary key
SensoiName	VARCHAR(20)	Sensor name	
Introduction	Text	Sensor introduction	

**Table 4:** Remote sensing clever image table.

Table name	Type of data	Annotation	Constraint
SensorID	INTEGER	Image automatic numbering	Primary key
SensorName	VARCHAR(20)	Image name	
	…		
FileLocation	VARCHAR(150)	File path	
MetadataID	INTEGER	Metadata number	Foreign key

**Table 5:** Metadata information table.

Table name	Type of data	Annotation	Constraint
MetadatalD	INTEGER	Metadata automatic numbering	Primary key
SatellitelD	INTEGER	Satellite number	Foreign key
SensorlD	INTEGER	Sensor number	Foreign key
Bands	VARCHAR(20)	Include band	
MapPrqjection	VARCHAR(50)	Projection method	
PixelSpacing	VARCHARQO	Spatial resolution	
TopLeftLong	FLOAT	Upper left longitude	
TopLeftLat	FLOAT	Upper left latitude	
TopRightong	FLOAT	Upper right longitude	
TopRightLat	FLOAT	Upper right latitude	

**Table 6:** Remote sensing images used in the research.

Satellite	Imaging date	Types of	Resolution (m)
Resource no. 3	2017.10.17	Panchromatic/multispectral	2.1/5.8
Gaofen no. 1	2019.11.09	Panchromatic/multispectral	2/8
Gaofen no. 1	2018.10.19	Panchromatic/multispectral	2/8
Gaofen no. 1	2020.08.22	Panchromatic/multispectral	2/8
Gaofen no. 1	2018.11.12	Panchromatic/multispectral	2/8

## Data Availability

The dataset can be accessed upon request to the corresponding author.
